# Gestational diabetes mellitus and impaired glucose tolerance pregnant women

**DOI:** 10.12669/pjms.306.5755

**Published:** 2014

**Authors:** Jinhua Wei, Jianbo Gao, Jinluo Cheng

**Affiliations:** 1 Jinhua Wei, MD, Department of Obstetrics, Changzhou Second People's Hospital. No 29 Xinglong Alley, Changzhou 21300, China.; 2 Jianbo Gao, MD, Department of Endocrinology, Changzhou Second People's Hospital. No 29 Xinglong Alley, Changzhou 21300, China.; 3 Jinluo Cheng, MD, Department of Endocrinology, Changzhou Second People's Hospital. No 29 Xinglong Alley, Changzhou 21300, China.

**Keywords:** Gestational diabetes, Gestational impaired glucose tolerance, Diabetes, BMI, Insulin resistance

## Abstract

***Objective:*** To evaluate correlations between insulin secretion and resistance in patients with gestational diabetes mellitus (GDM) and gestational impaired glucose tolerance (GIGT).

***Methods:*** Three hundred thirty six pregnant women with an oral glucose tolerance test (OGTT) were tested and measured insulin function indices (IFI), insulin resistance indices (HOMA-IR) as well as blood serum triglycerides (TG), total cholesterol (TCH) and low density lipoprotein cholesterol (LDL-C) concentrations. GIGT patients were further divided into subgroups according to hyperglycemia appearance 1, 2 or 3 hours after glucose ingestion.

***Results:*** GDM and GIGT correlated with age (p<0.05), family history of diabetes (p<0.05) and pre-pregnancy body mass indices (BMIs) (p<0.05). Blood pressures were higher in GDM than in GIGT and normal glucose tolerance (NGT) patients (p<0.05). The IFIs were gradually reduced (p<0.05), whereas HOMA-IR was gradually enhanced (p<0.05) in the GIGT and GDM patients. Blood serum TG, TCH and LDL-C concentrations were higher in the GIGT and GDM groups (p<0.05) and the GIGT 1 hour hyperglycemia subgroup had highest pregnancy weight gain and HOMA-IR values (p<0.05).

***Conclusions*** *:* Advanced age, family history of diabetes, high BMIs and blood pressure were risk factors for GIGT and GDM, which were both caused by reduced insulin secretion and enhanced insulin resistance.

## INTRODUCTION

Gestational diabetes mellitus (GDM) is kind of abnormal glucose tolerance during pregnancy^1^ and the prevalence is higher in western countries,^2^ but increasing year by year also in Asia,^3^^,^^4^ while the frequency of recurrent GDM in subsequent pregnancies is up to 50%.^5^ Early studies suggested that the pathogenesis of GDM is a defect of islet beta cell functions and insulin resistance. Insulin resistance refers to a decrease in the biological and physiological response to insulin, resulting in over secretion of insulin to compensate for the impairment of glucose transport into skeletal muscle and fat cells and inhibition of liver glycogen production. These changes can lead to a wide range of vascular lesions, resulting in small vascular endothelial cell proliferation and luminal narrowing, blood pressure increases and gestational hypertension. 

Caused by abnormal glucose metabolism blood and urine glucose concentrations are increased with concomitant enhanced susceptibility for ascending urinary tract infections. High blood sugar concentrations are transferred through the placenta into the fetal body, which leads to fetal growth stimulation and rising incidence of macrosomia.^6^^-^^8^ Hyperglycemia stimulates fetal islet B cell hyperplasia and hypertrophy with increased insulin secretion and high blood insulin concentrations. High insulin and high glucose causes embryonic hypermetabolic rates, which stimulate fetal peripheral hematopoietic increases and polycythemia neonatorum.^9^^,^^10^ After birth, the excessive glucose repletion is interrupted, which can result in neonatal hypoglycemia and might cause irreversible damage of brain cells.^10^

High insulin hematic diseases adversely affect corticosteroid metabolism, which in turn can promote the synthesis and release of fetal type 2 cells and the fetal lung maturity is delayed consequently.^11^ High insulin levels can also increase the risk of fetal distress and neonatal asphyxia, leading to the severe hypoxia of chemic encephalopathy.^9^^,^^10^ Endo et al. found that insulin sensitivity in obese women with normal glucose tolerance is lower than that in normal weight women and insulin sensitivity of pregnant women with GDM decreased with increasing gestational age.^12^ However, there are few reports about gestational diabetes and impaired glucose tolerance in China and especially research on predictors is rare, while recent studies reported significant ethical differences.^13^^,^^14^

In this study, we investigated prospectively the incidence of GDM and GIGT in Chinese pregnant women and analyzed insulin resistance as well as insulin secretion indexes and blood lipid correlations with glucose intolerance during pregnancy.

## METHODS

***Patients: ***From January 2012 to June 2013, 336 pregnant women in the obstetrics and the endocrinology departments of the Second People's Hospital, Changzhou City, Jiangsu Province were examined with a two-step diabetes-screening test. Exclusion criteria for this study were history of diabetes or impaired glucose tolerance (IGT), history of hypertension, high cholesterol, heart, liver and kidney diseases as well as history of autoimmune and other endocrine diseases before pregnancy. The research protocol was approved by the Ethics Committee of Changzhou Second People's Hospital and all patients gave written informed consent. The study met the Declaration of Helsinki and Good Clinical Practice.

**Diagnostic criteria**

***Two-step method of gestational diabetes screening: ***In the two-step OGTT, 50-gram glucose were initially ingested by the pregnant women and results were considered positive, if one hour later the plasma glucose value was higher than 7.8 mmol / L. The second step of the OGTT was done after 12 hours of fasting and 75-gram glucose powder dissolved in 200-300ml water was swallowed while venous plasma glucose concentrations before, one hour as well as 2 and 3 hours after glucose ingestion were analyzed. The common Chinese diagnostic criteria are fasting blood glucose of <5.6mmol / L and blood concentrations of <10.3mmol / L, <8.6 mmol / and <6.7 mmol / Lglucose 1, 2 and 3 hours after oral glucose uptake.^15^ If two or more values are above the diagnostic criteria limits, patients are diagnosed with GDM, whereas if only one value is higher, patients are diagnosed with GIGT. Finally, the participating pregnant women were categorized in NGT as well as GDM and GIGT groups. In this study, all GIGT cases were further divided into three subgroups, depending whether their plasma glucose concentrations were abnormal 1, 2 or 3 hours after glucose ingestion. Since there were only two cases with abnormal fasting blood glucose, we excluded them from the study (no fasting elevated plasma glucose group).

***Laboratory examination: ***We recorded age, gestational age, BMI, smoking history, family history of diabetes, and weight gain after pregnancy in addition to systolic blood pressure (SBP) and diastolic blood pressure (DBP), blood sugar as well as blood lipids of all participating pregnant women. During OGTTs, fasting insulin (FINS), TG, TCH and LDL-C data were collected. The homeostatic model assessment (HOMA)^16^ was used to calculate values of HOMA-insulin resistance (HOMA-IR), which are equal to fasting blood glucose (FBG) multiplied by fasting serum insulin (FINS) /22.5. Larger HOMA-IR values indicated more severe degree of insulin resistance. The islet β cell function was evaluated by using an insulin function index (IFI), which as calculated via FINS divided by FBG and larger IFI values indicated intact insulin metabolism.

***Statistical analyses: ***Data analyzes were done with the STATA 7.0 statistical software. Data of BMI, TG, TCH, LDL-C, ISI as well as HOMA-IR were all normally distributed and presented as mean ± standard deviation and were subjected to variance analyses. P values less than 0.05 were considered statistically significant.

## RESULTS

***Basic demographic data of the patients: ***We tested 336 pregnant women in total, among which NGT patients accounted for 51.8% (174 cases), GIGT patients accounted for 25.6% (86 cases) and GDM patients for 22.6% (76 cases). Table-I shows the clinical and biochemical characteristics as well as the results of the first step OGTT from all patients. By using pair wise comparisons, the data of one hour blood sugar levels after 50 gram glucose uptake in the first OGTT step were significantly higher in the GIGT and GDM groups compared to NGT patients (p <0.05). The patients in the GDM and GIGT groups were significantly older than in the NGT group (p < 0.05). The family history of diabetes was pronounced in the GDM (35.2%) and GIGT (24.1%) groups compared to the NGT (10%) patients (p<0.05). GMD and GIGT patients had significant higher BMIs than the NGT group (p<0.05). SBP and DBP were also significantly higher in the GDM compared to the GIGT and NGT groups (p<0.05) (Table-I). There was no significant difference regarding gestational age, pregnancy weight gain and smoking history among the groups. Our data suggest that hypertonic older patients with a family history of diabetes and high BMI values before pregnancy were at increased risk of developing glucose metabolism abnormalities during pregnancy.

***Metabolic parameters based on 75gram OGTT: ***Next, we evaluated the insulin secretion capacity of pancreatic beta cells in the different groups applying a 75-gram glucose OGTT after fasting for 12 hours. We found that the IFI values, which are indicating the secretion of pancreatic β cells, were highest in the NGT, followed by the GIGT and GDM patients with statistical significance between all groups ( p<0.05) indicating, that all pregnant women with impaired glucose tolerance had compromised islet β cell functions. The HOMR-IR value is associated with the degree of insulin resistance, which was gradually enhanced in the GIGT and GDM patients (p<0.05). Besides, TG, TCH and LDL-C values also varied from high to medium and low in the GDM, GIGT and NGT groups with statistical significance (p<0.05) (Table-II). These data indicated that GDM patients not only had declined islet β cell functions and increased insulin resistance, but also their lipid metabolism has undergone changes.

***Pregnancy weight gain and HOMA-IR correlated with OGTT hyperglycemia in the one hour GIGT subgroup:*** We divided the GIGT patients in subgroups, depending whether one blood glucose concentration was higher than 10.3mmol / L, 8.6 mmol / L or 6.7 mmol / L at 1, 2 and 3 hours after 75 gram oral glucose uptake. HOMA-IR and pregnancy weight gain values were significantly higher in the 1-hour hyperglycemia group, whereas all other parameter were not significantly different between the subgroups (Table-III). The data indicate that HOMA-IR abnormalities in the first hour of the OGTT can be used to determine GIGT.

## DISCUSSION

In this study, we diagnosed 86 GIGT (25.6%) and 76 GDM (22.5%) cases in 336 pregnant women and both correlated with age, BMI before pregnancy as well as family history of diabetes, while blood pressure was highest in GDM patients. This is in accordance with a recent report, which identified maternal age, elevated BMI, elevated DBP and family history of diabetes mellitus as significant risk factors for GDM.^17^ Also another study determined advanced maternal age, pre-pregnancy overweight or obesity and family history of diabetes as specific risk factors for GDM.^4^ We assessed the insulin metabolism unlike some other authors, which used the Insulin Secretion-Sensitivity Index (ISSI)^18^^,^^19^ by IFI and found that GDM and GIGT were gradually associated with impaired insulin secretion. Ryan et al. studied 14 women, who had a history of GDM and did not have signs of either diabetes or impaired glucose tolerance after birth, but in all of them serum glucose levels after oral glucose tolerance tests were significantly enhanced compared to the control group, which they attributed to a defect of insulin secretion.^20^ Also others described, that women with normal glucose tolerance and a history of gestational diabetes had significant impairments of beta-cell function at normal insulin sensitivity after birth.^21^

**Table-I:** Clinical characteristics of patients in the GDM, GIGT and NGT groups (n/%).

	**NGT**	**GIGT**	**GDM**	**p value**
Number of cases	174 (51.8%)	86 (25.6%)	76 (22.6%)	
Age (years old)	26.2±2.0^a^	29.3±3.2^b^	29.5±3.5^b^	< 0.05
Gestational weeks	26.1±1.6	26.2±1.7	26.2±1.8	
Pre pregnancy BMI (kg/m^2^)	21.2±3.2^a^	22.6±3.4^b^	22.6±3.5^b^	< 0.05
Pregnancy weight gain (kg)	7.5±2.9	7.5±2.7	7.5±2.8	
Systolic pressure (mmHg)	109.3±9.2^a^	109.1±9.1^a^	122.2±10.3^b^	< 0.05
Diastolic pressure (mmHg)	66.8±6.7^a^	66.9±6.8^a^	79.7±9.4^b^	< 0.05
One hour 50g OGTT blood glucose (mmol/L)	6.4±0.8^a^	9.0±1.1^b^	9.9±1.2^c^	< 0.05
History of smoking	4(2.3%)	2(2.3%)	2(2.6%)	
Family history of diabetes	17(9.8)%^a^	22(25.6%)^b^	27(35.5%)^c^	< 0.05

a, b and c indicate significant differences between groups.

**Table-II:** Metabolic parameters in the GDM, GIGT and NGT groups

	**NGT**	**GIGT**	**GDM**	**p value**
**IFI**	3.69±0.68^a^	2.92±0.45^b^	2.18±0.43^c^	<0.05
**HOMA-IR**	2.76±0.52^a^	4.39±0.72^b^	5.88±0.98^c^	<0.05
**TG (mmol/L)**	1.59±0.29^a^	2.67±0.58^b^	3.88±0.8^c^	<0.05
**TCH (mmol/L)**	3.91±0.66^a^	5.02±0.77^b^	6.14±0.93^c^	<0.05
**LDL-C (mmol/L)**	1.98±0.33^a^	2.58±0.45^b^	3.40±0.55^c^	<0.05

a, b and c indicate significant differences between groups.

**Table-III:** Clinical characteristics and metabolic parameters in GIGT subgroups.

	**1 hour**	**2 hours**	**3 hours**	**p-value**
Number of cases	24 (28.6%)	38 (45.2%)	22 (26.2%)	
Age (year)	29.3±3.1	29.3±3.3	29.3±3.1	
Gestational age	26.2±1.8	26.2±1.7	26.2±1.6	
Pre-pregnancy BMI (kg/m^2^)	22.6±3.2	22.6±3.4	22.6±3.5	
Pregnancy weight gain (kg)	8.5±2.9 a	6.9±2.7 b	7.0±2.8^b^	<0.05
Systolic blood pressure (mmHg)	108.3±9.0	109.1±9.1	108.5±9.2	
Diastolic blood pressure (mmHg)	66.9±6.7	67.0±6.8	67.1±6.9	
HOMA-IR	5.46±0.68 a	4.01±0.34^b^	3.92±0.33^b^	<0.05
TG (mmol/L)	2.64±0.54	2.69±0.60	2.64±0.63	
TCH (mmol/L)	4.98±0.67	5.04±0.77	4.99±0.72	
LDL-C (mmol/L)	2.58±0.55	2.57±0.54	2.59±0.49	

a and b indicate significant differences between groups.

 We also assessed insulin resistance with HOMA-IR in this study and found that HOMA-IR values in the GDM group were significantly higher than in GIGT or NGT patients, which indicated a significant insulin resistance. Studies carried out by Bartha et al. have shown that the insulin sensitivity index (ISI) in GDM patients was significantly lower than in non-affected pregnant women and the incidence of insulin resistance was significantly higher in GDM patients who needed insulin treatments.^22^ Our findings are in line with other reports, that GDM is caused by both reduced insulin secretion and enhanced insulin resistance^23^ and Saisho et al. suggested, that increased insulin resistance combined with beta cell dysfunction is associated with the severity of glucose intolerance and total insulin dosage required for GDM patients.^24^^,^^25^ The deficiencies might become manifest due to the women’s' hormonal changes during pregnancy, such as corticosteroids, human placental lactogen (HPL), estrogen and progesterone, placental enzymes, adiponectin^26^ and leptin.^27^

In our study, we found that the weight gain as well as the HOMA-IR were significantly higher in the 1-hour GIGT hyperglycemia than in the 2-hourand 3-hour subgroups, showing that a 1-hour glucose abnormality in GIGT patients was associated with higher insulin resistant severity. Recently, the International Association of Diabetes and Pregnancy Study Groups (IADPSG) revised the latest diagnostic criteria regarding hyperglycemia and adverse pregnancy outcomes. The new criteria were changed to one or more fasting, 1-hour or 2-hour plasma glucose concentration equal or higher than threshold values of 5.1, 10.0, or 8.5 mmol/L, respectively. The novel criteria are based on the classic tolerance test, but omit the 3-h blood plasma glucose value. 

Our finding, that 1-hour glucose abnormalities in GIGT patients were associated with more severe insulin resistance supports the latest criteria of IADPSG. Similar to the gradually reduced IFI and enhanced HOMS-IR changes in the GIGT and GDM groups we detected significant increases of TG, TCH and LDL-C serum concentrations (Table-II), which is supported by Chen et al., who noted that abnormalities in fat metabolism are present in both GDM and GIGT women.^28^ Because the fat storage increase and fat consumption reduce from the mid stage of pregnancy on, in advanced pregnancy stages the blood lipid also increase in parallel to regular physiological changes of increasing human placental prolactin levels with high lipolysis related to gestational weeks. Thus, changes in lipid metabolism may be a factor, which leads to physiological and pathological changes in women with GDM.^29^ In addition, free fatty acids were a key factor affecting islet cell function and insulin resistance in GDM patients.^30^ Our results also supported that GDM patients have lipid metabolism disorders, which may prelude GIGT in pregnant women.^31^

In summary, we found a high incidence of GIGT and GDM patients among the OGTT screened pregnant women, which correlated with their age, pre-pregnancy BMI and family history of diabetes while blood pressures were significant higher in the GDM group. The insulin functionality index was reduced and the insulin resistance was increased with ascending severity from the GIGT to the GDM group and with corresponding increases of total cholesterol, triglyceride and LDL-C serum concentrations.

## Authors Contribution:

**Jinluo Cheng: **Conceived, designed and did statistical analysis & editing of manuscript.

**Jinhua Wei and Jianbo Gao: **Did data collection and manuscript writing.

**Jinluo Cheng: **Takes the responsibility and is accountable for all aspects of the work in ensuring that questions related to the accuracy or integrity of any part of the work are appropriately investigated and resolved.
